# Chronicle of Hypoxemia: Transfusion-Associated Circulatory Overload Versus Transfusion-Related Acute Lung Injury

**DOI:** 10.7759/cureus.28712

**Published:** 2022-09-02

**Authors:** Shikha Jha, Keval V Patel, Amar Bukhari

**Affiliations:** 1 Internal Medicine, Saint Peter’s University Hospital, New Brunswick, USA; 2 Internal Medicine, Robert Wood Johnson Medical School, Rutgers University, New Brunswick, USA; 3 Cardiology, Robert Wood Johnson University Hospital, New Brunswick, USA; 4 Cardiology, Saint Peter’s University Hospital, New Brunswick, USA; 5 Pulmonary Critical Care, Saint Peter’s University Hospital, New Brunswick, USA

**Keywords:** hypoxemia, trali, taco, transfusion associated circulatory overload, transfusion related acute lung injury

## Abstract

The preeminent causes of blood transfusion-related morbidity and mortality are transfusion-associated circulatory overload (TACO) and transfusion-related acute lung injury (TRALI). These occur within hours of blood transfusion and lead to acute respiratory distress. The differentiation between TACO and TRALI has always been a great challenge in the context of underlying etiology, whether it is volume overload or lung injury, or both. This is a case report of a 64-year-old female with multiple comorbidities, who was brought to the emergency department with generalized weakness. She was hemodynamically unstable and encephalopathic. Her hemoglobin was 6.5 gm/dl with no active evidence of bleeding. She was started on a norepinephrine drip and one unit of packed red blood cells was transfused. A few hours post-transfusion, she became extremely tachypneic and hypoxic. A chest x-ray post-transfusion showed diffuse bilateral fluffy alveolar infiltrates and the N-terminal (NT)-pro hormone Brain Natriuretic Peptide (NT-proBNP) was significantly elevated. The transfusion reaction workup was negative. Due to worsening hypoxia, she required a rapid transition from non-invasive to invasive mechanical ventilation. The chronology of this case report depicts a unique presentation of acute respiratory distress and the course of hypoxemia.

## Introduction

Blood transfusion reactions are characterized by adverse reactions in response to transfusing whole blood or its individual components. They range in severity from minor to serious life-threatening reactions. They may present with nonspecific symptoms which usually overlap with other underlying comorbid conditions of the patients. This overlap of clinical presentations makes the diagnosis of transfusion reaction challenging. Mortality is not a common event in acute transfusion reactions but can occur rapidly [1]. Transfusion-associated circulatory overload (TACO) and transfusion-related acute lung injury (TRALI) are the prominent transfusion reactions leading to mortality. Both these conditions have common presenting features of hypoxemia and tachypnea. A thorough history, physical exam, close monitoring of event chronology, supporting lab, and imaging are required to differentiate the cause. 

## Case presentation

A 64-year-old female presented to the emergency department with generalized weakness for one week. She had associated lethargy, jaundice, and confusion. Her medical history was significant for heart failure with preserved ejection fraction, aortic stenosis status post valve replacement, and warm autoimmune hemolytic anemia. She recently completed steroid treatment and received a blood transfusion one week prior for warm autoimmune hemolytic anemia. On initial assessment, her vitals were pertinent for being hypotensive (blood pressure 92/58 mm Hg) with wide pulse pressure, mean arterial pressure (MAP) of 55 mm Hg, and tachycardic (heart rate 105 beats per minute), and regular, saturating 98% on room air. 

**Figure 1:**
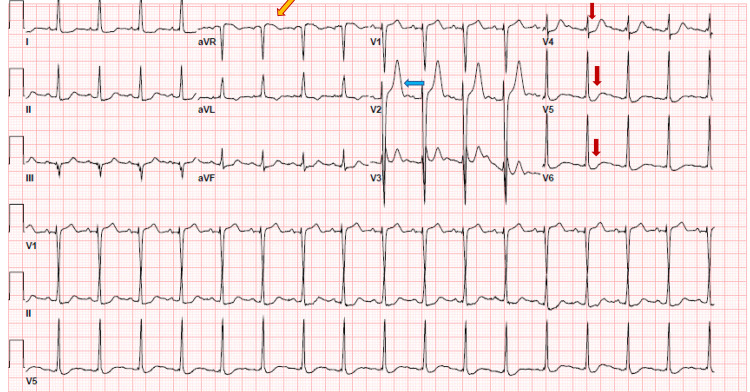
Electrocardiogram: Red arrows pointing towards ST depression in V4, V5, and V6: Blue arrow showing tall T wave; Yellow arrow showing aVR elevation

On physical exam, she appeared lethargic, oriented to self, bilateral icteric sclera, and lower conjunctival pallor present. On cardiac auscultation, she had an ejection systolic murmur in the right upper sternal border, grade 2, non-radiating. She had multiple spider nevi on chest inspection and bibasilar crackles on chest auscultation. Her abdominal exam was pertinent for distension, dullness on percussion, and shifting dullness. She had 1+ pitting edema in bilateral lower extremities. On skin exam, she had jaundice and decreased skin turgor. The electrocardiogram showed ST depression in V4, V5, and V6 lead indicating lateral ischemia, tall T waves on V2 lead, and aVR elevation in lead I indicating subendocardial ischemia (Figure 1). 

The pertinent lab investigations are shown in Table 1. The complete blood count with differential was pertinent for leukocytosis, anemia, macrocytosis, and thrombocytopenia. The hemolytic investigation showed elevated reticulocyte count and lactate dehydrogenase level, low haptoglobin level, and a positive direct comb test. The comprehensive metabolic panel showed impaired liver synthesis, indirect hyperbilirubinemia, and elevated liver-specific enzymes. The lactic acid level and ammonia levels were elevated. The kidney function was significantly impaired, and high anion gap metabolic acidosis was present. The cardiac troponin was remarkably elevated in the first set, then up trend in the subsequent values (Table 1).

**Table 1:** Pertinent lab investigation

Lab investigation	Values	Reference range
White Blood Cells	25.4 10 3/cu mm (High)	4.0-11.0 10 3/cu mm (cubic millimeter)
Hemoglobin	6.5 g/dL (Low)	12.0-16.0 g/dL (gram/deciliter)
Hematocrit	19 % (Low)	35.0-47.0 % (percent)
Mean Corpuscular Volume	103.5 fL (High)	80.0-100.0 fL (femtoliter)
Platelet count	54 10 3/cu mm (Low)	150-400 10 3/cu mm (cubic millimeter)
Reticulocyte count	12.6 % (High)	0.3-1.5 % (percent)
Lactate Dehydrogenase (LDH)	2241 U/L (High)	140-271 U/L (Unit per Liter)
Haptoglobin	<20 mg/dL (Low)	30-200 mg/dL (miligram/deciliters)
Direct Comb’s Test	Positive	
Albumin	2.8 g/dL (Low)	3.2-4.6 g/dL (gram per deciliter)
Prothrombin Time	39.6 seconds (High)	10.4-13.7 seconds
International Normalized Ratio (INR)	3.42 (High)	0.89-1.11
Total Bilirubin	12.5 mg/dL (High)	0.1-1.2 mg/dL (milligram per deciliter)
Indirect Bilirubin	7.5 mg/dL (High)	0.2-0.8 mg/dL (milligram per deciliter)
Alanine aminotransferase	100 U/L (High)	0-35 U/L (Unit per Liter)
Aspartate aminotransferase	272 U/L (High)	14-36 U/L (Unit per Liter)
Blood Urea Nitrogen (BUN)	67 mg/dL (High)	9-28 mg/dL (milligram/deciliter)
Creatinine	3.62 mg/dL (High)	0.52-1.04 mg/dL (milligram per deciliter)
Glomerular Filtration Rate (GFR)	13 ml/min/1.73 m2 (High)	>60 ml/min/1.73m2 (milliliters per minute per 1.73meter square) (For ages, 60-69 years)
Bicarbonate	15 mmol/L (High)	21-33 mmol/L (milimole per liter)
Anion Gap	15 mmol/L (High)	4 to 12 mmol/L (milimole per liter)
Lactic Acid	6.9 mmol/L (High)	0.5-2.0 mmol/L (milimole per liter)
Ammonia	60 micro mol/L (High)	16-53 micro mol/L (micromole per liter)
Cardiac Troponin 1^st^ set	4.3 ng/mL (High)	0.03 ng/mL (nanogram per militer)
Cardiac Troponin 2^nd^ set	5.38 ng/mL (High)	0.03 ng/mL (nanogram per militer)

**Figure 2:**
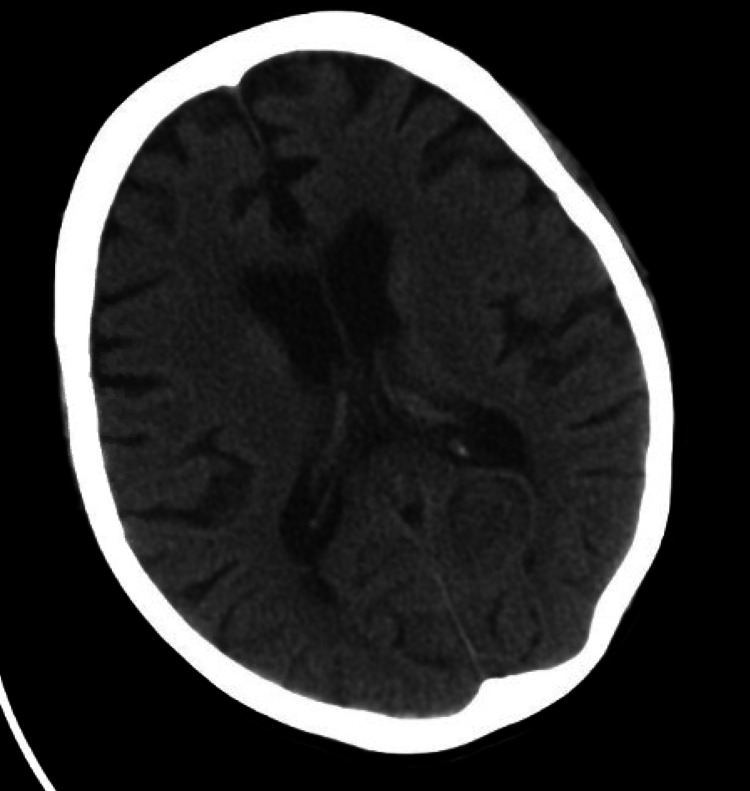
CT scan of the head: No acute intracranial abnormality

The CT scan of the head without contrast did not show evidence of acute intracranial abnormality [Figure 2].

**Figure 3:**
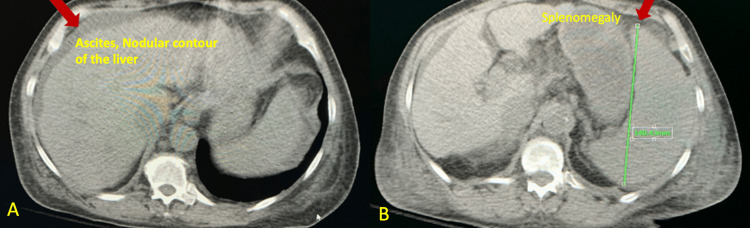
CT scan of the abdomen/pelvis - (A) Ascites and the nodular contour of the liver (B) Splenomegaly

The CT scan of the abdomen and pelvis without contrast showed an irregular contour of the liver, ascites, and splenomegaly (Figure 3).

**Figure 4:**
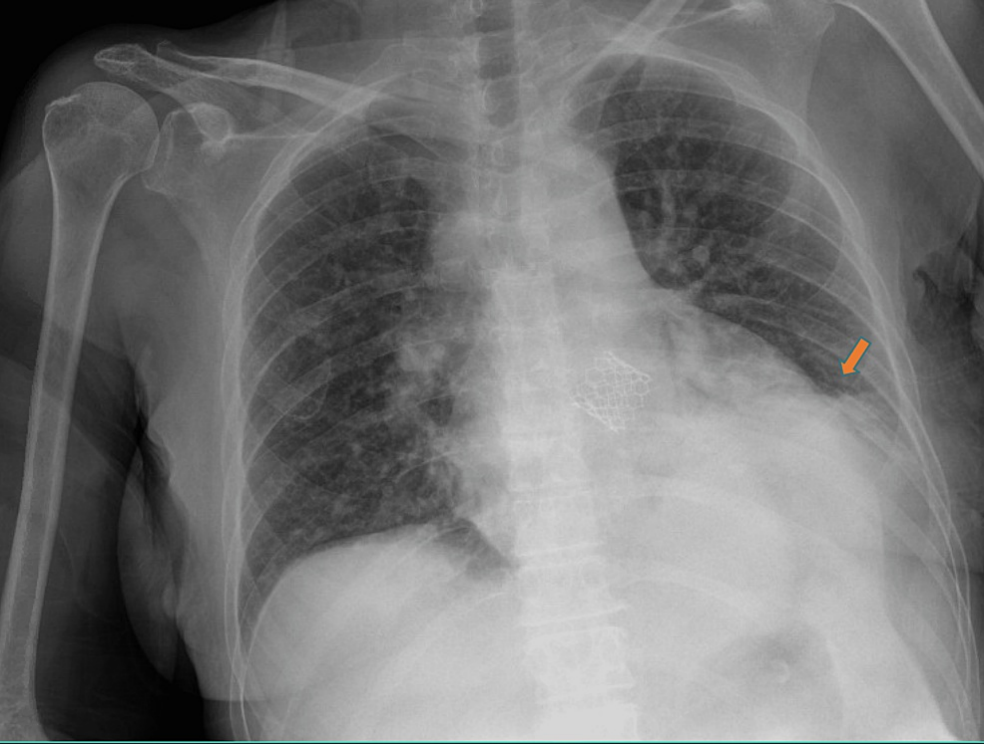
Chest x-ray Anteroposterior view - Left basilar opacity and elevation of the left diaphragm (indicated by the arrow)

The chest X-ray (Antero-Posterior view) showed patchy left basilar opacity and elevated left hemidiaphragm (Figure 4).

**Figure 5:**
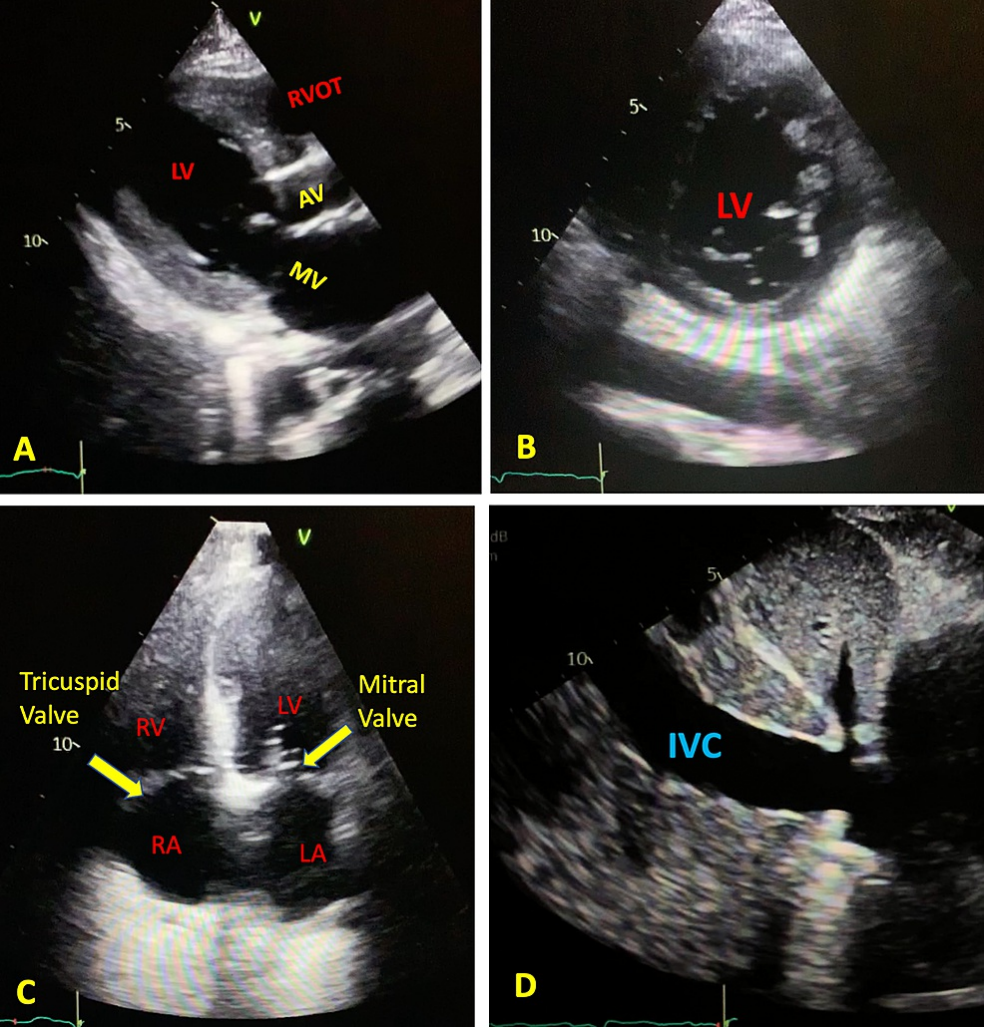
Cardiac Echo Cardiogram 2D - [A] Parasternal long axis view - LV [B] Parasternal short axis view [C] Four Chamber View [D] Subcostal view LA - Left Atrium, LV - Left Ventricle,  RA - Right Atrium, RV - Right Ventricle, AV - Aortic Valve, MV - Mitral Valve, RVOT - Right Ventricular Outflow Tract, IVC - Inferior Vena Cava

The patient had received intravenous fluid and albumin in view of severe hypotension. Transthoracic echocardiography was done after volume resuscitation. The different views are listed in Figure 5A-D. The echocardiography showed a left ventricular ejection fraction of 60%, grade I diastolic dysfunction, the transcatheter aortic valve in the aortic position, and good wall motion. Of note, the dilated inferior vena cava in the subcostal view is indicative of volume overload (Figure 5D). 

The patient had received a bicarbonate drip in view of metabolic acidosis, rifaximin due to hepatic encephalopathy, and antibiotics due to high suspicion of septic etiology. She was started on a norepinephrine drip for hypotension from shock. 

**Figure 6:**
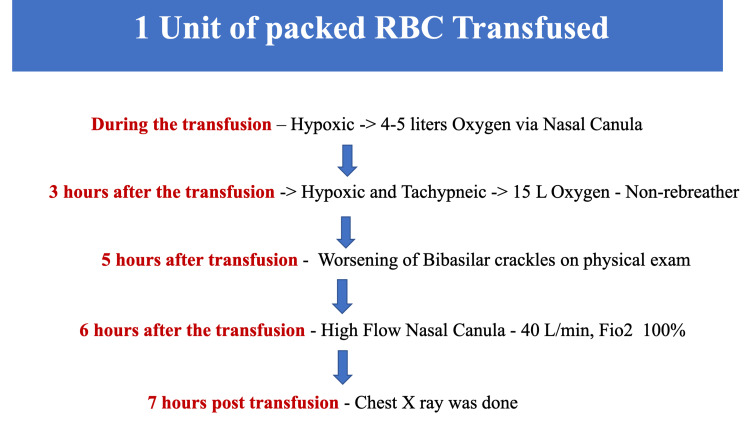
Flow chart: Chronology of events during and after blood transfusion L/min - Liter/minute, FiO2 - Fraction of inspired oxygen

In the meanwhile, 1 unit of packed red blood cells was transfused due to a low hemoglobin level of 6.5 gm/dl (gram per deciliter). During the transfusion, the patient became hypoxic, requiring 4-5 liters of oxygen via nasal cannula. After completion of transfusion, she became tachypneic and hypoxic, for which the oxygen requirement was advanced to 15 liters with non-rebreathers. The serial physical exam was significant for diffuse coarse crackles on chest auscultation, unlike the prior bibasilar crackles. The patient was put on a high-flow nasal cannula of 40 liters per minute and a FiO2 (fraction of inspired oxygen) of 100%. A flow chart describing the timeline of events is shown in Figure 6. 

**Figure 7:**
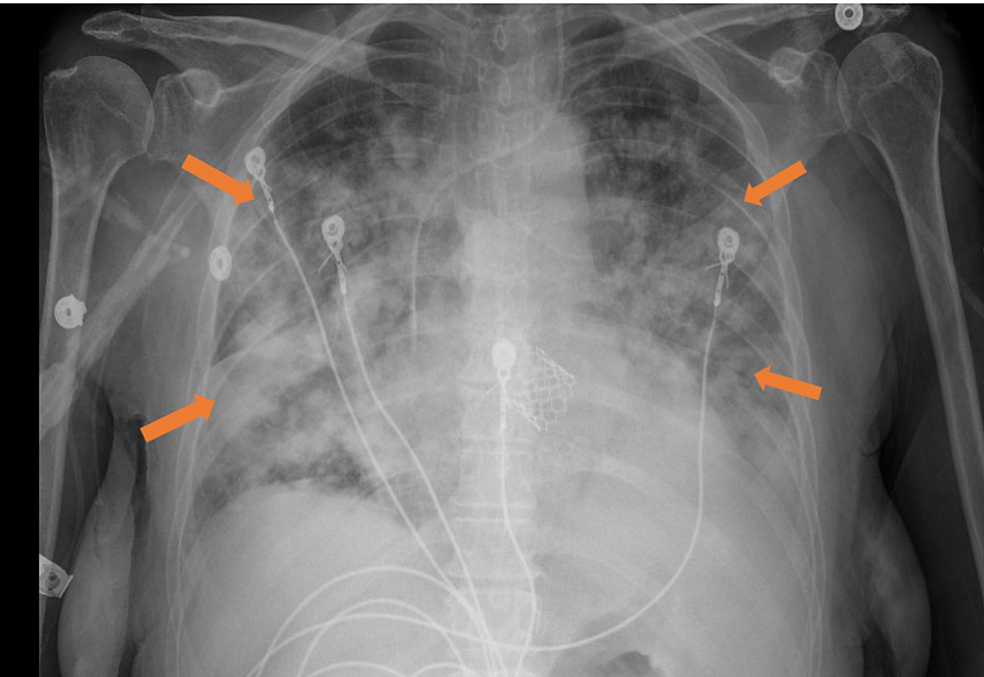
Chest x-ray 7 hours post-transfusion - Bilateral diffuse alveolar infiltrates (indicated by all the arrows)

A chest X-ray was ordered which now showed bilateral diffuse alveolar airspace opacities (Figure 7).

**Figure 8:**
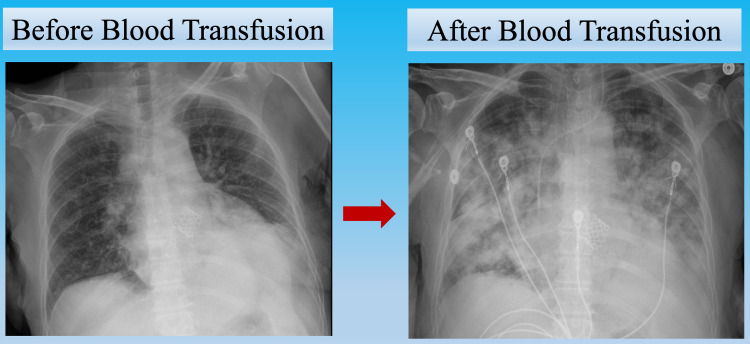
A comparison of chest X-ray: Pre- and Post- transfusion

An image comparing the initial chest X-ray to the post-transfusion chest X-ray is shown (Figure 8). 

An arterial blood gas analysis was done, which was nonspecific (Table 2).

**Table 2:** Arterial Blood Gas analysis

ABG analysis	Values	Reference range
pH	7.40	7.35-7.45
pCO2	32	32-45 mmHg (millimeter of mercury)
pO2	114	83-108 mmHg (millimeter of mercury)
HCO3	20	21-28 mmol/L (milimole per liter)
SpO2	99%	95-99 % (percent)

**Figure 9:**
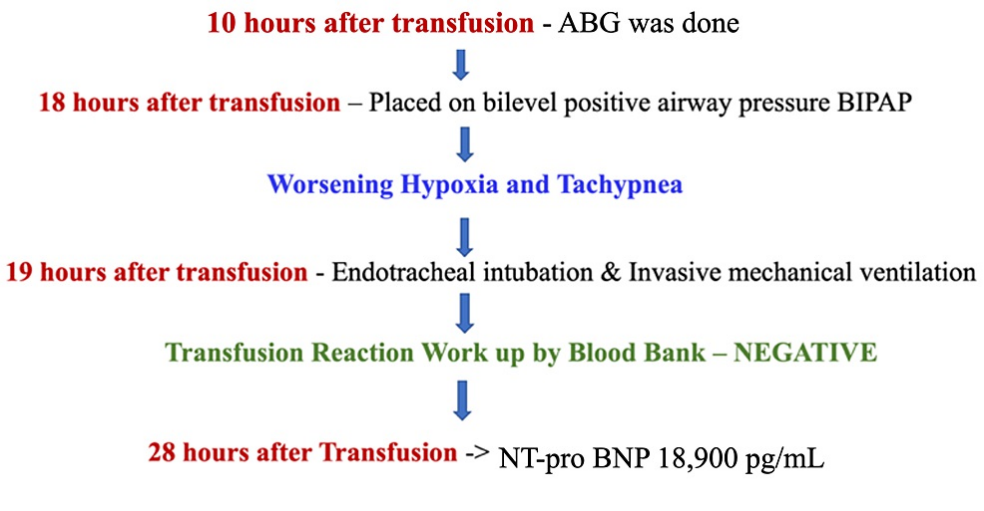
Flow chart: Chronology of events

Due to further worsening of oxygen saturation and respiratory rate, the patient then transitioned to bilevel positive airway pressure. Due to the rapid worsening of her clinical status, she was eventually intubated and placed on mechanical ventilation. The blood bank was notified. The subsequent transfusion workup was negative. At this time, an NT-pro-BNP (N-terminal pro B type Natriuretic Peptide) was done, which was significantly elevated at 18, 900 pg/mL (picogram per milliliter). A flow chart describing these timelines of events is shown in Figure 9. No additional fluids or further units of blood were administered. The patient was treated with full oxygenation support, diuretic, and close clinical and hemodynamic monitoring. The patient responded well to the diuresis with a decrease in oxygen requirement. 

## Discussion

Acute transfusion reactions may present with a variety of symptoms and signs during or within 24 hours of blood transfusion. These reactions may range from mild to severe dreadful consequences. The mild symptoms include fever, chills, urticaria, and pruritis, which resolve promptly without further need for treatment or complications. In severe cases, they may present with acute or worsening hypoxia, new onset tachypnea, loss of consciousness, hypertension, hypotension, flank or back pain, abnormal bleeding, oliguria, anuria, and jaundice which may be indicative of a more fatal reaction. Though mortality from a blood transfusion is a rare event, it is estimated to account for 0.6 per million to 2.3 per million [2]. While Acute Hemolytic Transfusion Reaction (AHTR) and TRALI were associated with the greatest deaths in the 2000s, the likelihood of fatality has now decreased. In the United States, TRALI was the most fatal blood transfusion reaction until 2016, following which TACO became the most fatal reaction. As per the reports from US Food and Drug Administration from 2013 to 2017, TACO accounted for the maximum number of deaths from transfusion reactions. On the other hand, TRALI was the second most common cause of maximum deaths in the same population. By convention, TACO and TRALI are the top differential diagnoses of each other in terms of blood transfusion reactions [3]. 

TACO manifests as pulmonary edema secondary to excess volume. Patients who develop TACO often have underlying renal or cardiovascular comorbidities. Symptoms develop within 12 hours of the blood transfusion. On physical exam, rales and third heart sounds are noted on auscultation. The chest x-ray shows perihilar vascular congestion, enlarged cardiac shadow, pleural effusion, septal lines, and interlobular fissure hypertrophy. As per the 2021 revised criteria by Biovigilance Network in the United States, definitive TACO is characterized by 3 or more of the following: respiratory distress, evidence of pulmonary edema on physical exam or imaging, and raised BNP or NT pro- BNP or other cardiovascular findings. The pathophysiology is believed to be caused by increased hydrostatic blood pressure that leads to leads to leakage of fluid in the alveolar space [4]. TACO is diagnosed by continuous clinical monitoring such as respiratory rate, oxygen saturation, blood pressure, and positive fluid balance. Chest imaging is helpful to detect pulmonary edema and rule out other differential diagnoses. The elevation of BNP or NT pro-BNP is helpful when combined with other clinical information [5]. 

It is important to distinguish TACO from other pertinent differential diagnoses, most importantly, TRALI. TRALI is caused by immune mediators such as anti-leukocyte antibodies in the transfusion component instead of volume overload. This leads to neutrophil activation, endothelial injury, and capillary leak leading to lung injury [6]. Both TACO and TRALI patients have similar presentations with acute hypoxia, dyspnea, rales on auscultation and diffuse bilateral infiltrates on chest x-ray. Unlike TACO patients, patients with TRALI may have a fever, hypotension, and transient leukopenia. The risk of TRALI is not secondary to the volume of transfusion as compared to TACO. TRALI patients become symptomatic during the transfusion or within 6 hours of completing the transfusion. 

As per the new TRALI definition proposed in 2019, TRALI has been classified into Type I and Type II. The TRALI Type I patient should not have risk factors for ARDS and should have the following criteria met: acute onset, hypoxemia (PaO2/FiO2 ≤300 mmHg* or SpO2 <90% on room air), bilateral pulmonary edema on imaging, no evidence of left atrial hypertension or, if is present, it is judged to not be the main contributor to the hypoxemia, onset during or within 6 hours of transfusion, and no temporal relationship to an alternative risk factor for ARDS. In TRALI type II, risk factors for ARDS are present. 

TRALI patients have nondistended neck veins on the exam, normal echocardiogram, neutral or negative fluid balance, exudative pulmonary edema fluid, and normal BNP, or NT pro-BNP levels [7]. Other differential diagnoses of TACO are acute hemolytic transfusion reaction, anaphylactic transfusion reaction, sepsis, febrile non-hemolytic transfusion reaction, and allergic transfusion reaction. Any transfusion reaction, whether TACO, TRALI, or other transfusion reaction should be reported to the transfusion service or blood bank for further evaluation. TACO is primarily managed by supportive care such as supplemental oxygen, and if needed, ventilatory support. Once the diagnosis of TACO is confirmed, the diuretic agent should be used for fluid mobilization. On the contrary, patients with TRALI require more fluid resuscitation for hemodynamic stability. 

To prevent transfusion reactions, a rapid transfusion rate should be avoided, and the volume of transfusion should be minimized as much as possible. Restrictive transfusion strategies aim at reducing such transfusion reactions. These strategies emphasize that transfusion should not be dictated by a hemoglobin “trigger” alone. Several landmark trials in transfusion medicine support the use of restrictive transfusion strategies. The Transfusion Requirement in Critical Care Trial, a large multi-center randomized, controlled trial compared restrictive (Hb <7 g/dL) to a liberal (Hb <10 g/dL) RBC transfusion strategy in ICU. This study showed a lower mortality rate during hospitalization in the restrictive group [8]. The Transfusion Requirement After Cardiac Surgery Trial is a large unit, prospective non-inferiority, randomized controlled trial, which compares a restrictive to a liberal RBC transfusion strategy in cardiac surgery. The restrictive group showed a non-inferior rate of combined 30-day all-cause mortality as compared to the liberal group [9]. The Functional Outcomes in Cardiovascular Patients Undergoing Surgical Hip Fracture Repair Trial is a large, randomized, controlled trial. It showed that a liberal transfusion strategy did not reduce rates of death or inability to walk independently on 60-day follow-up or reduce in-hospital morbidity in elderly patients at high cardiovascular risk, as compared to restrictive transfusion strategies [10]. The Choosing Wisely campaign from different societies emphasizes the importance of the wise use of blood transfusion with frequent reassessments and determining the absolute need for it [11]. 

## Conclusions

This case report depicts the challenges of differentiating between two major transfusion reactions, TACO, and TRALI, due to several overlapping features and preexisting comorbidities. Sound knowledge of the key differentiating features of these two blood transfusion reactions guides us towards appropriate diagnosis and management. Management of both these transfusion reactions primarily comprises prompt oxygenation and hemodynamic support. While diuresis remains the core management for TACO, on the other hand, fluid resuscitation continues to be the treatment approach for TRALI patients. To prevent further reactions, immediate reporting to the transfusion service is crucial. Also, the use of restrictive transfusion strategies can help mitigate future transfusion reactions. 
